# The Effects of the Explosion

**Published:** 1951-07

**Authors:** John H. Challenger

**Affiliations:** Consultant Anaesthetist, United Bristol Hospitals


### THE ATOMIC BOMB
### II: THE EFFECTS OF THE EXPLOSION

BY

JOHN H. CHALLENGER, M.B., Ch.B., D.A.
*Consultant Anaesthetist, United Bristol Hospitals*

---

The atomic bomb resembles bombs of the more conventional type in so far as the explosive effect is the result of the very rapid liberation of a large quantity of energy in a relatively small space. It differs however from other bombs in three important respects. First, the amount of energy released by an atomic bomb is a thousand or more times as great as that produced by the most powerful T.N.T. bombs. Secondly, the explosion is accompanied by highly penetrating and deleterious invisible rays, in addition to intense heat and light. Finally, the substances remaining after the explosion are radioactive, emitting harmful radiations.

The effects to be described are those that would be produced by the explosion of a " nominal " atomic bomb of the type dropped over Japan. Such a bomb has an effective energy release roughly equivalent to that produced by the explosion of 20,000 tons of T.N.T., and can be exploded in the air, under water or on the ground. Over this country the air burst is probably the optimum site for detonation, but the possibility of an under-water explosion with consequent widespread contamination by radioactive products carried in water droplets must not be overlooked. The bombs over Japan were exploded in the air at a height of about 2,000 feet. To obtain a comparable effect on a British city in which the buildings on the whole would be more resistant to heat and blast, the explosion would need to occur within a height range of from 500 to 1,000 feet.

When an atomic bomb is detonated in the air the first sign that anything has happened is a brilliant flash of bluish white light lasting a small fraction of a second. The light is accompanied by a flash of radiant heat which lasts a second or two after the intense brilliance is over, and which can ignite combustible materials up to a distance of two miles. At the same time the pressure wave is released and the hot source expands into a ball of fire several hundred feet in diameter. This ball of fire, surrounded for a few seconds by a ring of fog caused by a succeeding negative pressure wave, is then seen rushing upwards, expanding as it goes. It is followed by an opaque cloud of turbulent gases, and reaches the stratosphere in about four minutes. By this time the fire ball has disappeared and has been replaced by a mushroom-shaped cloud of white vapour some two miles in diameter.

The bulk of the radiation, consisting of alpha and beta particles, neutrons and gamma rays is emitted during the first second after the explosion. The alpha and beta particles have a range of only a few feet and may be neglected. Neutrons, which have a range of about 700 yards, are not likely to be a serious independent hazard, since anyone within that distance of an air burst would almost certainly be killed very quickly by blast, heat, or gamma radiation. We are left with gamma rays, which have an indefinite but very long range in air. In Japan some people were killed by them nearly a mile away from the burst.

Casualties produced by the air burst can be grouped under three headings:

1. Mechanical, due to blast, wounding by secondary missiles, and crushing by falling debris.

2. Thermal: flash burns, scorch burns and burns due to secondary fires.

3. Radiation:

(*a*) Produced by neutrons and gamma rays from the bomb.

(*b*) By radiation from radioactive substances.

1. *Mechanical.* The enormous pressure at the centre of the burst is dissipated as a blast wave. This pressure wave differs from that produced by an H.E. bomb. The blast wave from the latter passes in a few milliseconds and is succeeded by a period of feebler but more prolonged negative pressure during which most of the damage is done. Buildings tend to fall inwards towards the explosion. The blast from an atomic bomb takes a second or so to pass, and buildings are pushed outwards away from the explosion.

The secondary effects of blast, such as the collapse of buildings, flying debris and glass, are likely to be the most serious casualty-producing factors, resulting in fractures, lacerations and crush injuries. The fractures and contusions should not present any unusual features, but lacerations from flying glass may be more troublesome. In Japan they were often multiple with embedding of small fragments which were difficult to remove. Where radiation sickness developed slight wounds were said to break down and there was an increased tendency to gangrene.

2. *Thermal.* Two types of burns were observed at Hiroshima and Nagasaki. These were generally differentiated as (*a*) fire and flame burns, and (*b*) flash burns due to thermal radiations. Because of the numerous and widespread fires that develop after an atomic explosion, the total number of casualties, fatal and otherwise, due to flame burns will be very large. An atomic bomb releases roughly one-third of its total energy in the form of thermal radiation. For the " nominal " atomic bomb of the type we are considering, the energy emitted in this manner would be about $6{\cdot}7 \times 10^{12}$ calories, which is equivalent to about eight million kilowatt hours. It is evident that this enormous amount of energy, although acting only for a short time (about three seconds), would be expected to produce considerable damage.

The heat flash is capable of directly igniting inflammable material such as dark cloth, paper and dry wood, thereby starting many fires simultaneously over a wide area—under favourable atmospheric conditions up to a distance of two miles from the explosion centre. Clothing, particularly when white or light in colour, does afford protection against thermal radiations, and the majority of flash burns will be restricted to exposed parts of the body. A very high surface temperature is produced for a short time, and within the depths to which the thermal radiations penetrate the tissues appear to be completely destroyed. Marked redness of the skin appears almost immediately, with progressive darkening and blistering taking place over a period of a few hours. Uninfected first degree burns that are not irritated heal promptly, and keloid formation is not a special feature of the flash burn. For practical purposes of diagnosis and treatment it is not necessary to distinguish between burns caused by flame or by thermal radiation. In British cities two factors are expected to reduce the incidence of flash burns among people in the open. The weather is seldom clear over a city, and even a moderate amount of haze or mist causes a substantial reduction in the range of the thermal radiations. The second factor is that buildings provide a good measure of shielding, particularly in the centre of a city where streets are narrow and buildings high.

3. *Nuclear Radiation.* The injurious effects of radiation from the explosion of an atomic bomb represent an aspect completely absent from the conventional bomb burst. It should be clearly understood, however, that this does not imply that radiation is the most important source of casualties in an atomic explosion. In Japan, a maximum of 15 per cent. of the fatalities were attributed to nuclear radiation, compared with over 50 per cent. due to flame burns and blast, and more than 20 per cent. due to heat flash.

The effects of ionizing radiation on living organisms depend not only on the total amount absorbed, but also on the rate of absorption, and on the area of the body exposed. As far as the effects of an atomic bomb are concerned, the situation is simplified by the fact that the initial nuclear radiations are emitted for a short period so that exposure may be regarded as being of the acute type. On the other hand, residual radiations, due to fission products contaminating soil or materials, would represent a chronic hazard. Most of the victims of initial nuclear radiations in Japan were exposed over a large part of their bodies, since clothes are no protection against gamma rays. The cases reported have been roughly divided into three groups:

Those receiving:

(*a*) A lethal dose, *i.e.* 600r or more, which is fatal in nearly all cases within two weeks.

(*b*) A median lethal dose, *i.e.* about 400-500r, resulting in death to 50 per cent. in from two to twelve weeks.

(*c*) A moderate or sublethal dose, *i.e.* 100-300r which is generally not fatal.

### The Acute Radiation Syndrome

Following exposure to a dose of several thousand roentgens death may occur in a few hours, but there are few reliable observations on such cases. In cases of exposure to lethal but not extreme doses, varying degrees of shock appear within a few hours accompanied or followed by nausea and vomiting. Diarrhoea and fever of stepladder character begin during the first one or two days, with almost complete disappearance of the leucocytes. Scattered petechiae, and increasingly severe diarrhoea progress to sudden death in from four to ten days after exposure.

After exposure to a median lethal dose, nausea, vomiting, loss of appetite and malaise appear within a few hours. After the first day or two, all symptoms disappear and a latent period occurs, lasting up to two weeks, during which time the patient feels relatively well. During the third week there is a recurrence of symptoms which increase in severity with stepladder fever, bloody diarrhoea, loss of hair and ulceration about the lips extending throughout the alimentary tract in the terminal stages.

There is an immediate lymphopenia, followed by marked depression of the levels of all the white cell elements, due to destruction of bone marrow and lymphatic tissues. There is also a fall in the red corpuscle and platelet count. The blood count reaches its lowest level after about a week and then, in patients who are recovering, begins to increase. Where the symptoms are most severe death occurs within three to twelve weeks after exposure. Those who survive for three to four months and do not succumb to lung diseases or other complications gradually recover.

After exposure to a sublethal dose, there may be a latent symptom-free period up to two weeks or more: when the usual symptoms of anorexia, malaise, loss of hair, diarrhoea and tendency to bleed appear, but are not very severe. Typical changes in the blood picture are found, but their severity and persistence is much less marked. Without complications recovery is uneventful.

### Prognosis

It is clear that cases in Group 2 are those in which expert treatment is likely to be the deciding factor between death and recovery. Differentiation between the three groups at an early hour after admission to hospital is most important.

1. The personal dosimeter if carried will give valuable information, or failing this, the reading of an area dosimeter sent in with the casualty will help.

2. Blood examination. In the early stages the lymphocyte count is the most reliable guide. If the lymphocytes are below 200 per c.cm. after twenty-four hours the prognosis is probably hopeless. If above 1,000 per c.cm. after three days then the injury is probably trivial. Borderline cases may be expected to give counts between 300 and 500 per c.cm. after two days. At the end of the first week the total white count will be valuable. Experience has shown that those with fewer than 800 W.B.C. per c.cm. usually die, and those with more than 1,500 W.B.C. per c.cm. usually recover.

3. Fever is to be expected at some time, but a step-ladder climb in the first five days is of grave significance.

4. Gastrointestinal symptoms. Nausea and vomiting occur early as symptoms of both lethal and median dosage. Vomiting and bloody diarrhoea in the first three days is a sign that more than a median lethal dose has been received, and the prognosis is correspondingly grave. Where the casualty has received a median lethal dose or slightly less, the nausea, vomiting and malaise are less severe, and usually cease after twenty-four to forty-eight hours. Where the blood picture does not agree with the symptoms of fever and gastrointestinal disturbance, other causes, such as influenza or paratyphoid fever must be excluded.

5. Epilation: early and complete epilation is a serious sign, but not necessarily of fatal significance.

### Treatment of Radiation Casualties

Treatment resolves in the main to the group of casualties exposed to a median lethal dose (400—500r) of radiation and can be considered in three phases.

*First Phase.* During the first few days the patient may be regarded as being in a state of shock, requiring routine treatment with complete physical and mental rest, and conservation of body heat. Water balance must be watched, with intake and output records, and parenteral fluid given if there is evidence of dehydration. Care must be taken in forcing fluid or food by mouth because of local damage to the gut. Appetite is likely to return, and a low-residue high-protein diet, rich in vitamins, is indicated. Owing to the necrosis of lymphatic and haemopoietic tissues there may be a release of histamine-like substances: to counteract the effect of these antihistamine drugs are worth trying. Suprarenal cortical extract, given in 5 mgm. doses at eight-hour intervals, has been reported to be of some value.

*Intermediate Phase.* This is the period of negative symptoms, but positive signs, and the watchword during this phase is prophylaxis against infection. Good nursing is of paramount importance, for the prevention of pressure sores, and the maintenance of a clean mouth. The sulphonamide drugs are contraindicated. Prophylactic penicillin and streptomycin are good, but necessitate needle injection which in these cases is a potential source of skin infection. Aureomycin, which is effective against a wide range of bacteria and is given by mouth, is the antibiotic of choice. Blood, plasma, and plasma substitutes are not indicated at this stage. It is true that leucopenia exists, but whole blood transfusion is not the answer. The normal life of the leucocyte is only a matter of a day or so, so that the therapeutic effect, if any, would be transient. These therapeutic agents are likely to be in short supply, and are better reserved for normal traumatic casualties, where they are of known and proven value.

*Delayed Phase*. The earlier this phase appears, the poorer the prognosis. Death appears to be due either to haemorrhage or infection, but biochemical factors may also play a part. This is the time, if at all, for blood transfusion. Marrow failure means anaemia, and here it may be precipitated by haemorrhage due to thrombopenia and capillary damage. Stored blood will not provide platelets, but fresh blood, if available, may have a positive antihaemorrhagic action.

Toluidin blue, and protamine, given intravenously every other day while bleeding lasts, are said to be of some help. The flavonal glucoside Rutin, which is said to diminish capillary permeability, may be of value. Pentose nucleotide, leucocyte concentrates and marrow transfusions have proved disappointing. Plasma transfusion may help to supply protein for nutritional purposes. The plasma substitute dextran, a complex polysaccharide, which is utilized in the tissues, appears to promise valuable help in emergency.

